# Synergistic Uric Acid-Lowering Effects of the Combination of* Chrysanthemum indicum* Linne Flower and* Cinnamomum cassia* (L.) J. Persl Bark Extracts

**DOI:** 10.1155/2017/9764843

**Published:** 2017-07-09

**Authors:** Young-Sil Lee, Eunjung Son, Seung-Hyung Kim, Yun Mi Lee, Ohn Soon Kim, Dong-Seon Kim

**Affiliations:** ^1^KM Convergence Research Division, Korea Institute of Oriental Medicine, 1672 Yuseong-daero, Yuseong-gu, Daejeon 34054, Republic of Korea; ^2^Institute of Traditional Medicine and Bioscience, Daejeon University, 62 Daehak-ro, Dong-gu, Daejeon 34520, Republic of Korea

## Abstract

*Chrysanthemum indicum* Linne flower (CF) and* Cinnamomum cassia* (L.) J. Persl bark (CB) extracts have served as the main ingredients in several prescriptions designed to treat hyperuricemia and gout in traditional Chinese and Korean medicine. However, little is known about the combination effects of a CF and CB (CC) mixture on hyperuricemia. In our study, we investigated the antihyperuricemic effects of CC mixture and the mechanisms underlying these effects in normal and potassium oxonate- (PO-) induced hyperuricemic rats. The CC mixture significantly decreased uric acid levels in normal and PO-induced hyperuricemic rats and showed the enhanced hypouricemic effect compared to CF or CB alone. Furthermore, the CC mixture increased renal uric acid excretion in PO-induced hyperuricemic rat. We found that CC mixture and its major components, chlorogenic acid, 3,4-dicaffeoylquinic acid (isochlorogenic acid), coumarin, cinnamaldehyde,* trans*-cinnamic acid, and* o*-methoxycinnamaldehyde, inhibit the activity of xanthine oxidase (XOD) in vitro. The CC mixture exerts antihyperuricemic effects accompanied partially by XOD activity inhibition. Therefore, the CC mixture may have potential as a treatment for hyperuricemia and gout.

## 1. Introduction

Hyperuricemia is a metabolic disease characterized by elevated blood uric acid levels [1], the prevalence of which has increased worldwide. Hyperuricemia results from increased production or impaired excretion of uric acid [2, 3], and elevated uric acid levels cause accumulation of urate crystals in joints and the kidneys, leading to gout and gouty arthritis [4, 5]. It was also recently shown that hyperuricemia is associated with hyperlipidemia, hypertension, cardiovascular diseases, and diabetes [6]. Therefore, uric acid level regulation may play an important role in the prevention and treatment of various diseases, including hyperuricemia.

Uric acid is a final product of purine catabolism and is produced via the catalytic activities of xanthine oxidase (XOD), a rate-limiting enzyme responsible for converting hypoxanthine to xanthine, which is subsequently converted to uric acid [7]. In humans, seventy percent of the uric acid in the body is excreted on a daily basis by urate transporters, including the urate-anion transporter and organic anion transporter in the kidneys [8]. Accordingly, inhibiting uric acid biosynthesis and increasing uric acid excretion may be useful therapeutic approaches for the treatment of hyperuricemia. Currently, XOD inhibitors (allopurinol (AP) and febuxostat) and uricosuric agents (benzbromarone and probenecid) are used as antihyperuricemia drugs in clinical practice [9]. However, allopurinol and benzbromarone are poorly tolerated and reportedly induce side effects, such as drug allergies, renal failure, and hypersensitivity syndrome. These adverse effects limit the use of these drugs [10–13]. Thus, new antihyperuricemia agents are needed. Currently, natural products, such as herbal medicines, have emerged as new therapies that can overcome these limitations [13] and the numbers of studies regarding traditional herbal plants are increasing.

To identify herbal plants with antihyperuricemic effects, we screened hundreds of herbal medicinal plant extracts and found that* Chrysanthemum indicum* Linne flower and* Cinnamomum cassia* (L.) J. Persl bark extracts exert uric acid-lowering effects in animal models in a preliminary study.* Chrysanthemum indicum* is a traditional medicinal plant that has been used to treat inflammation-related diseases, several infectious diseases, and hypertension in Korea and China. Additionally, its flowers are used as herbal teas for the treatment of eye diseases and headaches and have various biological properties, including antioxidation and antitumor properties [14, 15].* Cinnamomum cassia *bark, which is also known as cinnamomi cortex, has been used in traditional medicine to treat immune-related diseases, gastritis, diarrhea, and cancer and is also a popular spice worldwide [16, 17].* Cinnamomum cassia* extracts also exhibit anticancer and antidiabetic effects [18, 19]. It has been reported that* C. indicum* flower and* C. cassia* bark have served as the main ingredients in several prescriptions designed to treat hyperuricemia and gout in traditional Chinese medicine [20]. According to a recent report, in an in vitro study of traditional Chinese medicinal plants, methanol extracts of* C. indicum* flower and* C. cassia* bark inhibited XOD activity [21]. Oil extracts from* C. indicum* flower,* C. cassia *bark, and* C. osmophloeum* leaves reduced serum uric acid levels in potassium oxonate- (PO-) induced hyperuricemic animals [20, 22, 23]. These findings indicate that* C. indicum* flower and* C. cassia* bark have antihyperuricemic effects. However, the combined effects of* C. indicum* flower and* C. cassia* bark extracts have not been studied. Thus, in the present study, we investigated the antihyperuricemic effects of* C. indicum* Linne flower,* C. cassia* (L.) J. Persl bark, and their combination in PO-induced hyperuricemic rats and normal rats, as well as the mechanism underlying these effects.

## 2. Materials and Methods

### 2.1. Materials

Chlorogenic acid, coumarin, 3,4-dicaffeoylquinic acid, cinnamaldehyde,* trans*-cinnamic acid, and* o*-methoxycinnamaldehyde were purchased from ChemFaces (China), respectively. The purities of them were determined to be 98% by high performance liquid chromatography (HPLC) analysis. Acetonitrile, methanol, and water were purchased from J. T. Baker (Phillipsburg, NJ, USA). Trifluoroacetic acid, allopurinol, dimethyl sulfoxide, xanthine oxidase from bovine milk (0.09 units/mg solid), xanthine sodium salt, and phosphate buffer (pH 7.2) were obtained from Sigma-Aldrich (St. Louis, MO, USA).

### 2.2. Sample Preparation

Flowers of* C. indicum* Linne and barks of* C. cassia* (L.) J. Persl were purchased from Kwangmyungdang Medical Herbs (Ulsan, South Korea). Flowers of* C. indicum* Linne, called “Indian* Dendranthema*” in English, were obtained at Zhejiang, China, and barks of* C. cassia* (L.) J. Persl, called “cinnamon” in English, were obtained at Yen Bai, Vietnam, respectively.* C. indicum* flower (CF) and* C. cassia *bark (CB) were identified by the Classification and Identification Committee of the Korea Institute of Oriental Medicine (KIOM) and their voucher specimens (numbers GHP-030 and 078) were stored at the herbarium of the Department of Herbal Resources Research of KIOM. They were ground and refluxed with 70% (*v/v*) ethanol for 3 hours using a Soxhlet extractor. Filtered extracts were concentrated using an evaporator (EYELA, Tokyo Rikakikai, Tokyo, Japan) at 45°C and then dried with a freeze dryer (Il-Sin Programmable Freeze Dryer-20R, Dongducheon, Korea) for 3 days. A total of 35.2 g of extract was obtained from 400 g of dried CF, and 31.0 g of extract was obtained from 400 g of dried CB for yields of 8.82 and 7.76%, respectively. Mixed samples were prepared from combinations of CF and CB extracts at weight ratios of 1 : 1, 1 : 2, 1 : 4, 2 : 1, and 4 : 1 to yield the following mixtures: CC11, CC12, CC14, CC21, and CC41. The samples were dissolved in 100% dimethyl sulfoxide to a final concentration of 50 mg/ml for in vitro efficacy testing and dissolved in methanol to a final concentration of 10 mg/ml for HPLC analysis. Each sample subjected to HPLC analysis was filtered using 0.45 *μ*m syringe filters (Millipore, Billerica, MA, USA).

### 2.3. Animals

Male SD rats (7 weeks) were purchased from Orient Bio (Seongnam, Korea) and were maintained at a temperature of 22 ± 1°C in a 50 ± 10% humidity-controlled room under a 12 h light/12 h dark cycle. The rats were allowed free access to a laboratory diet and water. The experimental design was approved by the Committee on Animal Care of the KIOM, and all experiments were performed in accordance with committee guidelines.

### 2.4. Sample Treatment in Normal Rats

The rats were randomized into the following 9 groups (*n* = 8/group) based on body weight: (1) a normal control group (NC), (2) a CF group, (3) a CB group, (4) a CC11 group, (5) a CC12 group, (6) a CC14 group, (7) a CC21 group, (8) a CC41 group, and (9) an AP group. The rats of NC group were treated with vehicle (0.5% CMC solution). CF, CB, and various CC mixtures in 0.5% CMC solution were given to the rats via oral gavage at a dose of 200 mg/kg. Allopurinol in 0.5% CMC solution was administered at a dose of 10 mg/kg.

### 2.5. Hyperuricemia Induction and Sample Treatment

For hyperuricemia induction, the uricase inhibitor potassium oxonate (PO) was administered as previously described [24, 25], with slight modifications. For the experiment regarding the effects of CF, CB, and various CC mixtures, the rats were divided into the following 10 groups (*n* = 8/group) based on body weight: (1) a normal control group (NC), (2) a hyperuricemia control group (PO), (3) a PO + CF group, (4) a PO + CB group, (5) a PO + CC11 group, (6) a PO + CC12 group, (7) a PO + CC14 group, (8) a PO + CC21 group, (9) a PO  +  CC41 group, and (10) a PO  +  AP group. The rats in groups (2)–(10) were injected intraperitoneally with 250 mg/kg PO prepared in 0.5% CMC with 0.1 M sodium acetate (pH 5.0) to induce hyperuricemia except group (1) that received 0.5% CMC with 0.1 M sodium acetate. Groups (1) and (2) received vehicle (0.5% CMC) by oral gavage, and groups (3)–(9) received CF, CB, CC11, CC12, CC14, CC21, and CC41 at a dose of 200 mg/kg. Group (10) was treated with allopurinol at a dose of 10 mg/kg. For the experiment examining the dose-dependent effects of the CC12 mixture, the rats were divided into the following 6 groups: (1) an NC group, (2) a PO group, (3) a PO  +  100 mg/kg CC12 group, (4) a PO  +  200 mg/kg CC12 group, (5) a PO  +  400 mg/kg CC12 group, and (6) a PO + AP group. All test samples were administered orally 30 min prior to PO injection.

### 2.6. Analysis of Uric Acid and Creatinine Levels in Serum and Urine

Urine samples were collected during 2 h after the final administration experiment and centrifuged (3000*g*, 10 min, 4°C) to remove particulate contaminants, and the supernatants were stored at −80°C until analysis. Blood samples were collected via cardiac puncture under anesthesia 2 h after urine collection following PO injection. Serum was obtained via centrifugation at 3000*g* for 10 min at 4°C after allowing the blood samples to clot for 2 h at room temperature. The separated serum was stored at −80°C until analysis. Serum and urine uric acid (SUA and UUA, resp.) levels were determined by an enzymatic-colorimetric method using commercial assay kits (Biovision, USA) according to manufacturer's protocols. Serum and urine creatinine levels were analyzed by an autoanalyzer (Hitachi-7020, Hitachi Medical, Japan). Fractional excretion of uric acid (FEUA) was calculated by the formula FEUA (%) = (urine  uric  acid  levels [mg/dl] × Serum  Creatinine  levels [mg/dl])/(serum  uric  acid  levels [mg/dl] × urine  creatinine  levels [mg/dl]) × 100.

### 2.7. Instrument Conditions for HPLC Analysis

To analyze the extracts, Agilent 1200 series instruments, including a degasser, binary pump, autosampler, and photodiode array detector, were used. Chromatographic separation was performed on a binary solvent manager, sample manager, column heater, and photodiode array detector with a Thermo Acclaim™ Polar Advantage C18 column (4.6 × 250 mm, 5 *μ*m) at a flow rate of 1.5 ml/min. The column oven was set at 40°C. The mobile phase consisted of 0.1% trifluoroacetic acid buffer (A) and acetonitrile (B). The gradient elution conditions were as follows: 10-10% B for 0–5 min, 10–23% B for 5–20 min, 23–25% B for 20–30 min, 25-25% B for 30–50 min, 25–50% B for 50–65 min, 50–100% B for 65–70 min, 100-100% B for 70–75 min, 100–10% B for 75–80 min, and 10-10% B for 80–85 min. The final sample consisted of 10 *μ*l of each sample, and the monitoring detector was set at a wavelength of 255 nm.

### 2.8. In Vitro XOD Inhibition Assay

Inhibition of XOD activity was analyzed based on decreases in uric acid formation at 295 nm at 37°C using a spectrophotometer. The reaction mixture comprised 50 mM sodium phosphate buffer (pH 7.6), 17.9 nM xanthine sodium salt, and 0.04 units of xanthine oxidase, and the reaction was started by adding xanthine oxidase and xanthine. Test samples were dissolved in DMSO (final concentration 0.1%, v/v) to final concentrations appropriate for the tested dose. XOD activity was compared with that of allopurinol, which served as a positive control. All the experiments were performed in triplicate, and IC_50_ values are expressed as the means of three experiments [26].

### 2.9. Statistical Analysis

Data are presented as the mean ± SEM. Differences among the treatment groups were analyzed by one-way ANOVA and Dunnett's multiple comparison test was applied to identify the significance using Prism 7.0 software (GraphPad Software Inc., San Diego, CA, USA), and *p* < 0.05 was considered statistically significant.

## 3. Results

### 3.1. Effects of CF, CB, and Various CC Mixtures on Serum Uric Acid Levels in PO-Induced Hyperuricemic Rats and Normal Rats

**Figure 1:**
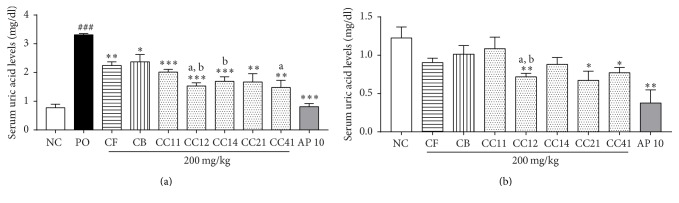
Effects of CB and CF and their combination on serum uric acid levels in PO-induced hyperuricemic rats (a) and normal rats (b). NC, normal control group; PO, potassium oxonate-induced hyperuricemia group; CF, 200 mg/kg* Chrysanthemum indicum* Linne flower ethanol extract; CB, 200 mg/kg* Cinnamomum cassia* (L.) J. Persl bark ethanol extract; CC, mixture of CF and CB; AP 10, 10 mg/kg allopurinol. Data are expressed as the mean ± SEM (*n* = 8). ^###^*p* < 0.001 versus the NC group; ^*∗*^*p* < 0.05, ^*∗∗*^*p* < 0.01, and ^*∗∗∗*^*p* < 0.001 versus the PO group; ^a^*p* < 0.05 versus the CF group; ^b^*p* < 0.05 versus the CF group.

The effects of CF, CB, and various CC mixtures on serum uric acid levels in PO-induced hyperuricemic rats and normal rats are shown in Figure 1. Serum uric acid levels in the PO group were significantly higher than those in the NC group (*p* < 0.001). Treatment with CF and CB at a dose of 200 mg/kg significantly reduced serum uric acid levels by 32% and 28%, respectively, compared with the PO group (*p* < 0.01, *p* < 0.05). Additionally, administration of various CC mixtures (CC11, CC12, CC14, CC21, and CC41) at a dose of 200 mg/kg significantly reduced serum uric acid levels by 45–55% compared with the PO group (*p* < 0.001, *p* < 0.001, *p* < 0.001, *p* < 0.01, and *p* < 0.01, resp., Figure 1(a)), and 10 mg/kg AP reduced serum uric acid levels by 75% (*p* < 0.001, Figure 1(a)) and exhibited hypouricemic effects similar to those noted in the NC group. Moreover, treatment of CC12 and CC41 mixtures significantly reduced the serum uric acid levels compared with the CF group and CC12 and CC14 mixtures showed lower serum uric acid levels compared with the CB group in PO-induced hyperuricemic rats (*p* < 0.05, all). As shown in Figure 1(b), CF and CB treatment decreased serum uric acid levels in normal rats, although this effect was not significant. Three 3 CC mixtures, CC12, CC21, and CC41, significantly decreased serum uric acid levels by 42%, 45%, and 40%, respectively, compared with the NC group (*p* < 0.01, *p* < 0.05, and *p* < 0.05, resp.), while the CC11 and CC14 mixtures had no effect on serum uric acid levels. Treatment with AP decreased serum uric acid levels by 70% (*p* < 0.01) compared with the NC group. CC12 mixture reduced the serum uric acid levels compared with the CF and CB groups in normal rats (*p* < 0.05). In this study, we found that CC mixtures exert better antihyperuricemic effects than CF or CB alone and that the CC12 mixture elicited a 55% reduction in uric acid levels, which was greater than that induced by the other CC mixtures. Thus, we selected the CC12 mixture for further study.

### 3.2. Dose-Dependent Effects of the CC12 Mixture on Serum and Urine Uric Acid Levels in PO-Induced Hyperuricemic Rats

**Figure 2:**
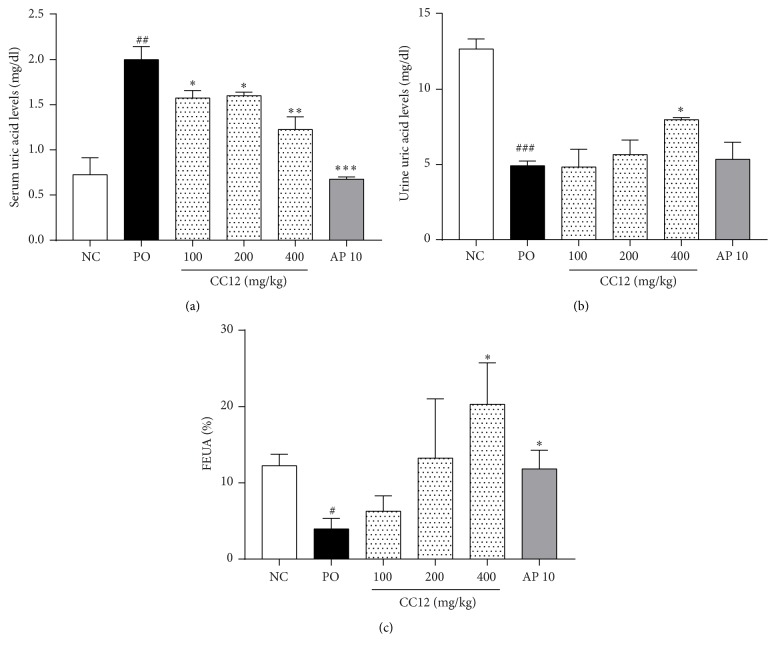
Dose-dependent effects of the CC12 mixture on serum (a), urine (b), uric acid levels, and fractional excretion of uric acid (FEUA) (c) in PO-induced hyperuricemic rat. NC, normal control group; PO, potassium oxonate-induced hyperuricemia group; CC12, mixture of CF and CB at a ratio of 1 : 2; AP 10, 10 mg/kg allopurinol. Data are expressed as the mean ± SEM (*n* = 8). ^##^*p* < 0.01 and ^###^*p* < 0.01 versus the NC group; ^*∗*^*p* < 0.05, ^*∗∗*^*p* < 0.01, and ^*∗∗∗*^*p* < 0.01 versus the PO group.

Serum uric acid levels were significantly higher in the PO group than in the NC group (*p* < 0.05). Treatment with the CC12 mixture at doses of 100, 200, and 400 mg/kg significantly reduced serum uric acid levels by 21%, 20%, and 39%, respectively, compared with the PO group (*p* < 0.05, *p* < 0.05, and *p* < 0.01, resp.). Additionally, 10 mg/kg AP treatment decreased serum uric acid levels by 66% (Figure 2(a)). Urine uric acid levels were reduced in the PO group compared with the NC group (*p* < 0.001). In contrast, treatment with 400 mg/kg CC12 mixture significantly increased urine uric acid levels by 1.6-fold (*p* < 0.05). 10 mg/kg AP treatment did not affect urine uric acid levels (Figure 2(b)). FEUA was lower in the PO group than in the NC group. CC12 mixtures showed the tendency to increase the FEUA and 400 mg/kg CC12 mixture treatment significantly increased by 5-fold compared with the PO group (*p* < 0.05, Figure 2(c)), although there was no significant change in serum and urine creatinine levels among treated groups (data not shown). 10 mg/kg AP also increased the FEUA (*p* < 0.05, Figure 2(c)).

### 3.3. HPLC Analysis of the CC12 Mixture and Content Analysis of Its Components

**Figure 3:**
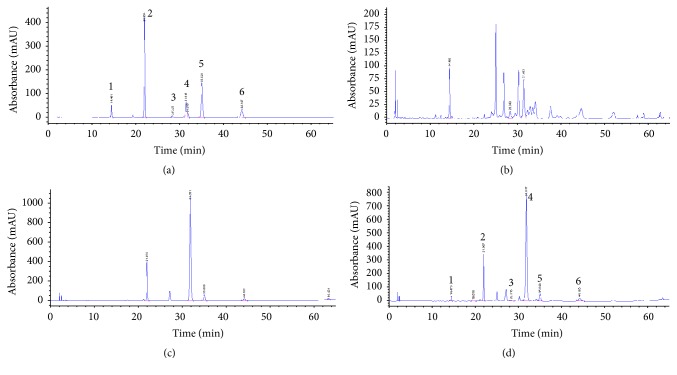
Representative HPLC chromatograms of a standard solution (a), CF (b), CB (c), and the CC12 mixture (d): (1) chlorogenic acid, (2) coumarin, (3) 3,4-dicaffeoylquinic acid, (4) cinnamaldehyde, (5)* trans*-cinnamic acid, and (6)* o*-methoxycinnamaldehyde.

HPLC chromatograms of CF, CB, and the CC12 mixture are shown in Figure 3. Their chemical components were identified from UV spectra and retention times compared with reference materials. Four compounds, coumarin, cinnamaldehyde*, trans*-cinnamic acid, and* o*-methoxycinnamaldehyde, composed CB, and two compounds, chlorogenic acid and 3,4-dicaffeoylquinic acid (isochlorogenic acid), composed CF. The representative HPLC chromatogram of the CC12 mixture showed three major components, chlorogenic acid, coumarin, and cinnamaldehyde, and their contents were 0.55, 3.70, and 11.84%, respectively, as shown in Table 1.

### 3.4. Effects of the CC12 Mixture and Its Components on XOD Activity In Vitro

Table 2 shows the effects of the CC12 mixture and its components on XOD activity. CF inhibited XOD activity by 39.2% at a concentration of 500 *μ*g/ml, showing weaker effects than its counterparts. CB inhibited XOD activity by 31.2%, 100.3%, and 98.4%, respectively, at concentrations of 100, 250, and 500 *μ*g/ml. The CC12 mixture also inhibited XOD activity at concentrations of 100, 250, and 500 *μ*g/ml by 23.2, 94.3, and 101.0%, respectively. We next investigated the components of CF and CB. The IC_50_ values of chlorogenic acid and 3,4-dicaffeoylquinic acid (CF) were 103.8 *μ*g/ml and 79.7 *μ*g/ml, respectively. The IC_50_ values of coumarin, cinnamaldehyde,* trans*-cinnamic acid, and* o*-methoxycinnamaldehyde (CB) were 95.2 *μ*g/ml, 43.7 *μ*g/ml, 58.8 *μ*g/ml, and 25.7 *μ*g/ml, respectively.

## 4. Discussion

Hyperuricemia is a metabolic disorder characterized by an imbalance between uric acid production and excretion and causes gout. The prevalence of hyperuricemia has increased worldwide in recent decades [27]. Currently, only limited numbers of drugs are available for the treatment of hyperuricemia, and many of these agents have adverse effects. Therefore, more effective antihyperuricemia agents are needed.

In this study, we examined the effects of CF, CB, and their combination on hyperuricemia. We found that CF, CB, and various CC mixtures significantly decreased serum uric acid levels in PO-induced hyperuricemic rats. Our results correspond to those of previous reports demonstrating that* Chrysanthemum* flower oil and* Cinnamomum* bark and leaf oils reduced serum uric acid levels [20, 22, 23]. We also determined whether these agents exert allopurinol-like effects in normal rats. The CC mixtures reduced serum uric acid levels, while CF and CB did not affect uric acid levels in normal rats, indicating that CC mixtures can exert allopurinol-like hypouricemic effects. Moreover, these CC mixtures were more effective at decreasing serum uric acid levels than CF or CB alone, and the CC12 mixture exhibited the synergistic effects on serum uric acid levels in PO-induced hyperuricemic rats and normal rats. Additionally, the CC12 mixture significantly reduced uric acid levels in a dose-dependent manner in PO-induced hyperuricemic rats, although its antihyperuricemic effects appeared to be weaker than those of allopurinol. These results suggest that the combination of CF and CB can exert enhanced antihyperuricemic effects. As mentioned above, although there have been reports regarding the antihyperuricemic effects of CF and CB, this study is the first to demonstrate that a CC mixture, a combination of CF and CB, exerts synergistic antihyperuricemic effects. These results suggest that the CC mixtures, as well as CF and CB, exert hypouricemic effects and CC12 mixture exerts better antihyperuricemic effects than other CC mixtures, which may represent alternative treatments for hyperuricemia.

To elucidate the underlying mechanisms of the antihyperuricemic effects of the CC12 mixture, we evaluated the effects of the CC12 mixture on XOD activity. XOD is a limiting enzyme that catalyzes the oxidation of hypoxanthine to produce xanthine and the subsequent oxidation of xanthine to produce uric acid in purine catabolism [28, 29]. Because hyperuricemia results from uric acid overproduction and underexcretion, XOD inhibitors that can inhibit the production of uric acid have been considered for the treatment of hyperuricemia. Furthermore, it has been demonstrated that oils obtained from CF and CB can inhibit XOD in vitro [21, 23, 30, 31]. In our study, we found that the CC12 mixture showed the tendency to inhibit the liver XOD activity in PO-induced hyperuricemic rats, although this effect was not significant (data not shown). CB and CC12 exerted moderate inhibitory effects on XOD in vitro, while CF exerted very weak inhibitory effects. In addition, 6 components of the CC12 mixture, namely, chlorogenic acid and 3,4-dicaffeoylquinic acid from CF and coumarin, cinnamaldehyde,* trans*-cinnamic acid, and* o*-methoxycinnamaldehyde from CB, exerted inhibitory effects on XOD activity in vitro. According to Wang et al. [23] and Meng et al. [32], chlorogenic acid and cinnamaldehyde significantly decreased serum uric acid levels by inhibiting XOD activity in PO-induced hyperuricemic mice. Based on our results and previous reports, cinnamaldehyde may be the primary contributor to the inhibitory effects of CB and the CC12 mixture on XOD activity, as it is main constituent of these compounds (15.13% and 11.84%, resp.). In contrast, chlorogenic acid does not seem to be a major active compound because its content is too low (1.46%), and CF exerted weaker inhibitory effects on XOD in vitro than its counterpart. In spite of its weaker inhibitory effects on XOD activity, CF was as effective at lowering serum uric acid levels as CB in PO-induced hyperuricemic rats, indicating that other mechanisms may underlie the hypouricemic effects of CF. Moreover, given that the CC12 mixture exerted relatively weaker inhibitory effects on XOD in vitro and strong uric acid-lowering effects in vivo, the synergistic effects of the CC12 mixture in vivo may also be attributable to other mechanisms. Thus, the beneficial antihyperuricemic effects exerted by the CC12 mixture in this study may be accomplished, at least in part, by its inhibitory effects on XOD, and its components may be responsible for its hypouricemic effects. However, further investigation is needed to elucidate the other mechanisms underlying the synergetic effects of the mixture.

Uric acid excretion as well as uric acid production plays an important role in uric acid homeostasis [2, 3]. We further examined whether the CC12 mixture could increase renal uric acid excretion via analysis of urine uric acid levels and FEUA, renal uric acid handling parameter. FEUA is used as marker of proximal tubular handling of uric acid and is defined as the percentage of uric acid excreted to creatinine excreted by glomeruli which is finally excreted in the urine. Therefore, increased FEUA can be interpreted as the increased excretion of renal uric acid. In the present study, uric acid levels in urine and FEUA reduced in the PO group compared to the NC group although there are reports demonstrating the uricosuric effect of PO [33, 34]. This observation is in agreement with finding of various reports [33, 34]. This discrepancy might be associated with the differences in approaches for administration periods and collecting time of urine. Interestingly, high doses of the CC12 mixture significantly increased urine uric acid levels and FEUA in PO-induced hyperuricemic rats. Previous report demonstrated that* Chrysanthemum* flower oil accelerated the uric acid excretion by upregulation of genes expression for uric acid transporters in kidney [22]. Based on this reporting, hypouricemic effect of CF might be related to enhanced uric acid excretion, which might be contributed to the synergistic effects of CC12 mixture. Our result suggests that CC12 mixture has ability to promote the renal uric acid excretion, which may be effect on reduced serum uric acid levels. However, additional studies are needed to analyze renal and urine uric acid levels of CF, CB, and their combination as well as the expression of renal organic anion transporters associated with urate handling.

## 5. Conclusions

The present study demonstrated that the CC12 mixture reduced serum uric acid levels in normal and PO-induced hyperuricemic rats and that its effects were stronger than those of its components, CF and CB, alone. Moreover, the CC12 mixture and its compounds inhibited XOD activity in vitro. Thus, the antihyperuricemic effects of the CC12 mixture may be due, at least in part, to its inhibitory effects on XOD activity, although further study is necessary to determine its effects on renal uric acid excretion. Based on these results, we propose that the CC12 mixture exerts beneficial effects on hyperuricemia and may be useful in the treatment of hyperuricemia.

## Figures and Tables

**Table 1:** Contents of marker compounds in CC12 mixture.

Contents (%)	CF	CB	CC12 mixture
Chlorogenic acid	1.46		0.55
3,4-Dicaffeoylquinic acid	0.46		0.16
Coumarin		4.24	3.70
Cinnamaldehyde		15.13	11.84
*trans*-Cinnamic acid		0.38	0.33
*o*-Methoxycinnamaldehyde		1.1	1.55

CF, *Chrysanthemum indicum* flower ethanol extract; CB, *Cinnamomum cassia* bark ethanol extract; CC, mixture of CF and CB.

**Table 2:** XOD activity of CF, CB, and CC12 mixture and their marker compounds.

Sample treatment	Concentration (*μ*g/ml)	% inhibition	IC_50_ (*μ*g/ml)
CF	100	5.3 ± 2.8	>500
	250	8.9 ± 4.1	
	500	39.2 ± 6.9	
CB	100	31.2 ± 2.6	143
	250	100.3 ± 3.1	
	500	98.4 ± 0.1	
CC12 mixture	100	23.2 ± 0.4	153.8
	250	94.3 ± 3.2	
	500	101.0 ± 0.2	
Chlorogenic acid	62.5	24.0 ± 1.7	103.8
	125	63.6 ± 1.5	
	250	107.3 ± 0.6	
3,4-Dicaffeoylquinic acid	62.5	21.0 ± 1.5	79.7
	125	104.6 ± 2.6	
	250	85.0 ± 1.2	
Coumarin	62.5	22.8 ± 0.1	95.2
	125	75.1 ± 1.0	
	250	103.9 ± 0.7	
Cinnamaldehyde	62.5	101.2 ± 3.2	43.7
	125	126.3 ± 5.6	
	250	118.8 ± 35.1	
*trans*-Cinnamic acid	62.5	54.3 ± 0.7	58.8
	125	96.7 ± 1.9	
	250	114.9 ± 0.4	
*o*-Methoxycinnamaldehyde	62.5	104.7 ± 0.2	25.7
	125	101.2 ± 0.2	
	250	100.0 ± 0.4	
Allopurinol	6.25	51.7 ± 0.7	
	12.5	71.7 ± 0.1	
	25	80.3 ± 0.3	

*Chrysanthemum indicum* Linne flower ethanol extract; CB, *Cinnamomum cassia* (L.) J. Persl bark ethanol extract; CC, mixture of CF and CB. Data are expressed as the mean ± SEM.
